# A Clinicopathological Categorization System for Clinical Research in Coccidioidomycosis

**DOI:** 10.1093/ofid/ofad597

**Published:** 2023-11-29

**Authors:** Paul Krogstad, George R Thompson, Arash Heidari, Rasha Kuran, Alexis V Stephens, Manish J Butte, Royce Johnson

**Affiliations:** Department of Pediatrics, David Geffen School of Medicine at UCLA, Los Angeles, California, USA; Department of Molecular and Medical Pharmacology, David Geffen School of Medicine at UCLA, Los Angeles, California, USA; Division of Infectious Diseases, UC Davis School of Medicine, Sacramento California, USA; Department of Medicine, David Geffen School of Medicine at UCLA, Los Angeles, California, USA; Dignity Health, Bakersfield Memorial Hospital, Bakersfield, California, USA; Department of Medicine, David Geffen School of Medicine at UCLA, Los Angeles, California, USA; Department of Medicine, Kern Medical, Bakersfield, California, USA; Institute of Precision Health, University of California, Los Angeles, California, USA; Department of Pediatrics, David Geffen School of Medicine at UCLA, Los Angeles, California, USA; Department of Microbiology, Immunology, and Molecular Genetics, David Geffen School of Medicine at UCLA, Los Angeles, California, USA; Department of Medicine, David Geffen School of Medicine at UCLA, Los Angeles, California, USA; Department of Medicine, Kern Medical, Bakersfield, California, USA

**Keywords:** coccidioidomycosis, dissemination, meningitis, pathology, valley fever

## Abstract

A wide array of clinical manifestations follow infection with *Coccidioides immitis* or *Coccidioides posadasii*, ranging from asymptomatic infection to life-threatening pulmonary disease or extrapulmonary dissemination and meningitis. Epidemiological studies require consistent definitions of cases and their comparative clinical features. Understanding host and pathogen determinants of the severity of coccidioidomycosis also requires that specific clinical features (such as coccidioidal meningitis) and their overlap be precisely defined and quantified. Here we propose a system for categorization of outcomes of coccidioidomycosis in individuals who are not overtly immunocompromised that harmonizes clinical assessments during translational research of this increasingly common disease.

## Teaser

The clinical manifestations of coccidioidomycosis range from asymptomatic infection to life-threatening pulmonary disease, extrapulmonary dissemination, and meningitis. We propose a system for categorization of outcomes of coccidioidomycosis to harmonize clinical assessments during translational research of this increasingly common disease.

Coccidioidomycosis (CM) is a systemic infectious disease caused by 2 species of thermally dimorphic fungi (*Coccidioides immitis* and *Coccidioides posadasii*) found principally in the Western United States, Mexico, Central America, and certain regions in South America. In the United States, the incidence of CM has been increasing since 2000, with a steady rise between 2009 and 2019 [1]. Factors thought to be responsible for this sustained increase include the expansion of suitable environmental conditions for the growth of *Coccidioides* in the soil and a surge in the population of major cities in the endemic region [2]. The documented increase in the number of cases and areas in which CM is now being found heightens the importance of better understanding the pathophysiology of this condition and improving on existing therapeutic approaches [3–6].

The 2 species of *Coccidioides* exhibit minor differences in their growth characteristics in vitro, but their life cycles and the clinical manifestations they generate are indistinguishable. Both species grow in filamentous form within the upper several centimeters of soil. Septation of fungal hyphae occurs during fungal growth and alternate cells undergo autolysis, liberating arthroconidia. These arthroconidia are dispersed when the soil is disturbed by wind, excavation, recreational activities, and other phenomena. Once inhaled into the lungs, the arthroconidia germinate and form spherules; internal segmentation of these spherules produces multiple endospores. Eventually, the spherules rupture and release these endospores, each of which has the potential to spread widely into adjacent tissue or disseminate via the bloodstream to extrapulmonary sites [7]. The physiological mechanisms for resisting progressive infection and/or dissemination are incompletely understood, as are the regenerative and homeostatic programs that allow for recovery from infection with or without residua.

As summarized by Galgiani and colleagues [4], coccidioidal infections exhibit a wide range in the severity of clinical outcomes that may be placed into 4 general categories (Table 1). Delayed-type hypersensitivity testing studies conducted in the mid-20th century in California demonstrated that the majority of coccidioidal infections (approximately 60%) are inapparent or unrecognized. Most other infections are associated with fever, cough, and other signs of pulmonary infection, and they may be accompanied by reactive skin lesions (erythema nodosum or erythema multiforme) [8, 9]. These infections are often indistinguishable from other lower respiratory tract infections caused by viral or bacterial pathogens. Evidence of this comes from a case-control study performed in Arizona in which 15% of individuals presenting with prolonged chest complaints or community-acquired pneumonia in 2003 and 2004 had serological evidence of CM [10].

**Table 1.** Traditional Categorization of Coccidioidal Infections

Category	Synonym	Characteristics
Asymptomatic	Inapparent infection	Prior infection recognized by delayed hypersensitivity testing or serological means
Uncomplicated pulmonary disease	…	Infection suspected and confirmed based on evidence of signs and symptoms and epidemiology
Complicated pulmonary disease	…	Include fibrocavitary disease, ranging from thin-walled cavities to areas of necrosis and phlegmon
Extrapulmonary dissemination	Extrathoracic dissemination	Evidence of infection outside the chest revealed by culture or histopathology

In a small fraction of coccidioidal infections, severe pulmonary disease occurs and necessitates hospitalization for management of hypoxia, pleural effusions, respiratory failure, and other acute complications. Furthermore, in an estimated 0.5% of cases, extrapulmonary dissemination of disease occurs. While any organ system can become involved, musculoskeletal, central nervous system (CNS) infections and granulomatous skin lesions are the most common manifestations of disseminated disease [4, 6, 8]. Meningitis is a dreaded complication; CNS cerebrospinal fluid shunting is needed in approximately half of cases, and death nearly always occurs in the absence of treatment with antifungal agents [4, 11, 12].

Studies in the late 20th century revealed that poorly controlled diabetes, human immunodeficiency virus (HIV) infection, and iatrogenic immunosuppression increase the risk of disseminated CM [11, 12]. In a small number of selected cases of disseminated disease, monogenic dysfunctional innate or cellular immune mechanisms have been identified [13]. However, most cases of disseminated disease occur in individuals without overt clinical evidence of immunodeficiency. Genetic studies of individuals with severe or disseminated disease have revealed that mutations in dectin-1 and downstream signaling involved in detecting cell wall components of fungi or in activating the production of hydrogen peroxide are found in a large fraction of individuals with disseminated CM [6, 14].

Subtle differences in CM clinical phenotypes have been noted that suggest that the simple classification depicted in Table 1 obscures underlying differences in pathophysiology that may have an impact on efforts to diagnose and treat the disease or that may complicate development of protective vaccines. For example, high serum concentrations of complement fixing antibody are seen in most cases of CM with extrapulmonary dissemination, but this elevation is less commonly seen in coccidioidal meningitis [7] (which represents sequestered disease in which serum antibody titers to *Coccidioides* do not correlate with the state of disease). Surprisingly, coccidioidal meningitis is less likely to be diagnosed in individuals with more extensive dissemination to locations outside the CNS, and those with monoarticular dissemination to the knee or hip joints are unlikely to concurrently have coccidioidal meningitis. These observations suggest that the immunological defenses that protect the host from dissemination to the CNS and other sites may differ. Grouping CNS dissemination alongside the spread of CM to other extrapulmonary sites simply as “disseminated disease” could impede recognition of critically important host-pathogen interactions. With these examples and other nuances of CM in mind, we propose a categorization scheme for CM in individuals without evidence of overt primary or acquired immunodeficiency states to be used in studies examining the interplay between *Coccidioides* fungi and infected individuals that determines the outcome of infection.

## METHODS

We used a version of the Delphi method [15] in which iterative discussions were followed by review of sequential drafts of the schema until consensus was reached. The physician participants are all board certified in infectious diseases (P. K., G. R. T., A. H., R. K., and R. J.) or allergy-immunology (M. J. B.) and practice at 3 regional referral centers for CM in the endemic area; A. V. S. is a clinical trials specialist. All have extensive experience in clinical or translational research in CM [6, 11, 12, 16]. We initiated this process with a round table discussion based on established general categorizations of clinical CM [4, 11, 17]. A written and graphic summary of a proposed schema was prepared and circulated among participants for a subsequent virtual meeting. This process was repeated for 2 additional cycles. The resulting schema is distinctly different from those used in the longitudinal monitoring of patients in clinical trials and provides a means of categorizing individuals undergoing immunological and clinical evaluation designed to clarify the pathophysiological basis for differences in outcomes of coccidioidal infection. Because this work did not involve the use of human subjects, patient consent was not required.

## CLINICOPATHOLOGICAL CATEGORIES OF CM

The following paragraphs and Table 2 outline a scheme for characterization of the end points of CM after respiratory tract exposure to *C immitis* or *C posadasii*.

**Table 2.** Clinicopathological Schema for Coccidioidomycosis

Clinical Features	Category^a^	Subcategory^a^	Details
No prior CM	0	0A	No prior coccidioidomycosis; may have traveled or lived in an area in which cases of CM are consistently found
		0B	Has never visited or lived in an area where CM is endemic
Unrecognized CM in the past	1	…	No clinical recognition of coccidioidal disease but evidence of prior infection
Uncomplicated pulmonary CMNo evidence of extrapulmonary dissemination	2	…	Proved or probable coccidioidal lung disease with or without antifungal therapy; may have chest radiographic evidence of effusion or parenchymal disease, lymphadenopathy
Complicated pulmonary CM	3	3A	Pulmonary disease with simple cavitation
No evidence of extrapulmonary dissemination		3B	Fibrocavitary lung disease
		3C	Persistent pulmonary disease for >6 mo despite therapy
		3D	Respiratory failure with or without multisystem organ failure
Extrapulmonary dissemination of CM without meningitis or other CNS involvement	4	4A	Extrapulmonary dissemination without meningitis; skin only
		4B	Extrapulmonary dissemination without meningitis to locations other than skin (eg, osteomyelitis, infective arthritis, or visceral organs)
Coccidioidal meningitis (or other CNS involvement of CM)	5	5A	Meningitis; no other evidence of extrapulmonary dissemination
		5B	Meningitis with additional dissemination to skin (only)
		5C	Meningitis with extrapulmonary dissemination to skin and other organs
Death attributable to CM	6	…	Death caused by CM

Abbreviations: CM, coccidioidomycosis; CNS, central nervous system.

^a^In category 0, CM is excluded by having a negative result with *Coccidioides* immunoglobulin G immunodiffusion (IgG-ID) serology or with delayed-type hypersensitivity skin testing using *Coccidioides* antigens. Individuals in category 1 must have a positive IgG-ID result with no history of a disease compatible with CM. Individuals may also be included who have proof of CM from histological identification of spherules as an incidental finding in pathological specimens. Category 2 is defined by uncomplicated pulmonary CM with no evidence of extrapulmonary dissemination. Individuals in this group must have a clinical diagnosis of pulmonary CM (eg, cough, chest pain, or sputum production) and proof of *Coccidioides* infection by ≥1 of the following: positive IgG-ID result; positive *Coccidioides* complement fixation test result; growth of *Coccidioides* in a culture of sputum, pleural fluid, or other intrathoracic tissue; or spherules (endosporulating spherical structures) visualized in pulmonary nodules, intrathoracic lymph nodes, or lung tissues. Note that pulmonary symptoms must last <6 months with no evidence of cavitation or fibrocavitary disease at chest radiography (or other chest imaging) performed during a follow-up visit. Category 3 includes complicated pulmonary CM. Must have proof of infection, as for category 2, along with 1 of the following subcategories: 3A, confirmation of prior *Coccidioides* infection and thin wall cavity identified by tissue examination or chest radiography; 3B, confirmed infection and characteristic radiological changes of cavitation and fibrotic interstitial markings or abscess formation; 3C, persistence or progression of pulmonary disease with or without antifungal therapy for ≥6 months, worsening chest imaging findings, or abnormal pulmonary function tests; or 3D, respiratory failure (oxygen saturation <96% while breathing air, arterial blood partial pressure of oxygen/fraction <70 mm Hg, or arterial blood oxygen pressure/fraction of inspired oxygen ratio <330), with or without multisystem organ failure and septic shock. Category 4 includes extrapulmonary dissemination of CM without meningitis or other CNS involvement. This category requires proof of infection, as for category 2, or growth of *Coccidioides* from body fluids or tissues outside the respiratory tract, and it also requires no signs, symptoms, or radiographic/CSF evidence of meningitis or other CNS complications of *Coccidioides* infection) (eg, arachnoiditis or hydrocephalus); lumbar puncture is *not* required to exclude CNS infection. Subcategory 4A includes cutaneous disease without meningitis—spherules demonstrated in tissue from a skin biopsy or growth of *Coccidioides* sp. in culture of a skin biopsy specimen; subcategory 4B, extrapulmonary dissemination to sites other than skin and without CNS disease; this subcategory requires visualization of spherules in extrapulmonary fluid or tissue other than skin or growth of *Coccidioides* from an extrapulmonary sample (except skin). Most commonly, this will involve osteomyelitis, extrapulmonary lymph nodes, or solid organs. Category 5 is defined by dissemination of *Coccidioides* infection to the CNS; this requires growth of *Coccidioides* from a cerebrospinal fluid (CSF) sample, or positive ID/complement fixation serological results in CSF with an abnormal CSF white blood cell count or a low CSF glucose level (hypoglycorrhachia). Subcategory 5A includes coccidioidal meningitis with no evidence of extrapulmonary dissemination to other tissues; subcategory 5B, coccidioidal meningitis with evidence of dissemination to skin (spherules and/or growth of *Coccidioides* from skin sample); and subcategory 5C, coccidioidal meningitis (as in category 4A) but with foci of extrapulmonary dissemination other than the skin (eg, bone or joints). Category 6 includes deaths attributable to CM but without sufficient information to determine whether extrapulmonary dissemination has occurred.

### No Evidence of Prior CM: Category 0

We propose a category (category 0) for individuals who have no evidence of anticoccidioidal antibodies by immunodiffusion (immunoglobulin M or G) or complement fixation assays or skin test evidence of delayed hypersensitivity reactions to coccidioidal antigens. This dependence on immunological methods necessitates careful exclusion of individuals with concurrent conditions (eg, people living with HIV), those receiving immunosuppressive medications following solid organ or hematopoietic stem cell transplantation), or those with prior disease states or treatments that may interfere with test sensitivity. CM is largely restricted to specific areas in North and South America and the risk of acquisition of infection increases with duration of exposure [8, 18]. We propose a subcategory based on geographic exposure to areas where cases of CM are consistently found (category 0A) for those who may have traveled or lived in the endemic area. Individuals who have never visited or lived in traditional endemic areas (category 0B) are less likely to have acquired coccidioidal infection and could constitute an appropriate control group in studies of clinical outcomes of CM. It is worth remarking that absence of proof of infection does not mean proof of absence of infection, and while we currently lack another approach to fully clear someone of infection, category 0 is a categorization of exclusion.

### Unrecognized Prior CM: Category 1

This classification (category 1) represents most cases of CM and comprises individuals with immunological, microbiological, or histopathological evidence of prior infection with *Coccidioides* without symptoms or mild illness that escapes clinical recognition. Unrecognized infection has been clearly demonstrated in studies in which delayed-type hypersensitivity reactions or humoral immunity have been systematically found in adults and children with defined periods of habitation in classic endemic areas [8, 18]. Incidental histopathological identification of spherules within tissue (eg, in tissue collected during evaluation of pulmonary nodules) has also demonstrated that inapparent or asymptomatic infection regularly occurs [19].

### Uncomplicated Pulmonary CM: Category 2

Individuals in this group (category 2) will have had a clinical diagnosis of pulmonary CM (eg, fatigue, cough, chest pain, sputum production, and/or fever [4, 8, 10]) with proved or probable *Coccidioides* infection by ≥1 microbiological, serological, or histopathological method (see footnote to Table 2). Consequently, uncomplicated disease refers to cases in which pulmonary symptoms last for ≤6 months with no evidence of cavitation or fibrocavitary disease in a subsequent chest radiograph. It is important to recognize that provisionally designating a case as category 2 necessitates a period of observation of 6 months, since evidence of pulmonary sequelae and disease progression (including extrapulmonary dissemination) may not appear for ≥4 months [8, 16].

### Complicated Pulmonary CM: Category 3

Category 3 comprises cases of pulmonary disease that are accompanied by the development of radiographically distinct residua, are particularly severe at the outset, or are protracted compared with self-resolving disease. The least complicated form of CM is complete recovery from symptomatic infection with ≥1 thin wall cavities identified by chest radiography (category 3A). Although these cystic lesions eventually resolve in most cases [20], these lesions may be further complicated by superinfection by bacteria or other fungal species. In some instances, fibrocystic changes (category 3B) persist indefinitely. Although these permanent changes have been said to be more common in persons living with diabetes mellitus with CM, this may reflect the facts that primary cystic diseases occur more commonly in the setting of diabetes mellitus [21] or that the inflammatory state described in association with diabetes allows more lesions to progress into more complex lesions [22].

Some individuals exhibit persistence (≥6 months) or progression of pulmonary disease (worsening chest imaging findings and abnormal pulmonary function) despite antifungal therapy, long after most cases of acute pulmonary disease resolve. Pulmonary hemorrhage, cavity rupture, and empyema may occur. The mechanisms that permit progression of disease in this group of individuals (category 3C) remain undefined. Provisional category 2 cases that fail to resolve all symptoms by 6 months since disease onset but that do not otherwise progress would fall into this persistent/progressive pulmonary category. Finally, we note that some individuals present with diffuse pneumonia and experience respiratory failure within the first 1–2 weeks after coccidioidal infection is first recognized (category 3D) [23]; in some cases, this is accompanied by multisystem organ failure.

### Extrapulmonary Dissemination of CM Without Meningitis or Other CNS Involvement:

#### Category 4

When CM disseminates beyond the lungs, it may do so only to skin (category 4A). It is identified in this scenario by demonstration of spherules and endospores in skin biopsy specimens or growth of *Coccidioides* in cultures of such specimens. By contrast, widespread dissemination may occur without spread to the CNS (category 4B). Most commonly, this clinical phenotype will involve osteomyelitis (involving ≥2 bones in approximately half of cases); extension into extrapulmonary lymph nodes, solid organs, or synovial joint spaces; or the formation of abscesses or phlegmons. Imaging studies, such as computed tomography, positron emission tomography/computed tomography, and bone scanning, are often performed to determine the extent of spread [16]. Category 4B does not require that each abnormal site on imaging studies have pathological confirmation of spread. Peritonitis may occur without clear identification of a specific intra-abdominal focus [24]. Importantly, dissemination is often recognized at a time when any prior respiratory tract symptoms have resolved.

#### Dissemination of *Coccidioides* Infection to the CNS: Category 5

Dissemination of coccidioidal infection to the spinal cord, brain, and meninges is infrequent, but the lethality of untreated CNS and the propensity of meningitis to relapse if antifungal therapy is stopped [11] are distinctive clinical features. Some cases of coccidioidal meningitis are without any evidence of extrapulmonary dissemination to other tissues (category 5A) or are accompanied only by skin lesions that can be proved to contain viable fungi (histopathological recognition of spherules or growth of *Coccidioides* from skin biopsy samples) (category 5B). By contrast, and much more rarely, coccidioidal meningitis with other extensive foci of extrapulmonary dissemination (eg, musculoskeletal foci, spread to internal organs) is also encountered (category 5C). Hypothetically, these 3 scenarios may reflect an incremental severity of disease, reflecting greater fungal replication, differences in immunological control of fungemia, or other mechanisms.

#### Death Attributable to CM: Category 6

On occasion, a diagnosis of CM is confirmed by premortem or postmortem serological, microbiological, or pathological studies but without sufficient information to determine the role that *Coccidioides* infection may have played in the individual’s death.

## DISCUSSION

We have proposed a system for classifying the most common outcomes of CM in ostensibly immunocompetent individuals based on our extensive experience with the range of CM’s clinical manifestations after acquisition of infection by respiratory exposure. We note that this schema does not address the clinical expression of CM following rare alternative routes of exposure, such as direct inoculation into skin (primary cutaneous CM) [9], or following donor-derived transmission of infection [25]. It must be emphasized that accurate classification requires sufficient longitudinal follow-up (4–6 months), since progression of disease and extrapulmonary dissemination after such an interval generally do not occur in individuals without evidence of inborn errors of immunity or generally recognized immunocompromising conditions or treatments.

For the purpose of investigating genetic or immunological factors that might influence the course of CM, we have proposed criteria for inclusion of individuals into control groups who are assumed to have no prior infection by *Coccidioides* fungi (category 0). We acknowledge that this approach is imperfect: the sensitivity of current immunological methods for detection of *Coccidioides* infection varies and is affected by the time since infection occurred. Recruitment of individuals who have never lived in traditional endemic areas within the Americas will reduce but not eliminate the possibility of prior infection. State of residency is not predictive by itself, given the potential for significant travel: 290 (1.4%) of the 20 003 case reports of CM received by the Centers for Disease Control and Prevention in 2019 originated from outside Arizona, California, Nevada, New Mexico, and Utah [3]. Similarly, a carefully detailed travel history does not fully exclude the possibility of exposure, in view of the recent evidence of expansion of *Coccidioides* beyond what were seen as the limited endemic areas [1, 2].

One potential limitation in using this proposed classification system is its dependence on microbiological, histopathological, and immunological detection methods for proving prior CM. Each of these diagnostic approaches has its technical limitations. Validated polymerase chain reaction assays to detect the fungal genome (or subgenomic DNA fragments), biochemical detection of fungal metabolites, and other diagnostic methods to improve diagnostic certainty would improve evaluation of the natural course of CM.

The classification system was devised with a version of the Delphi method [15]: a series of teleconferences were each followed by review of proposed drafts of the schema, until general consensus was reached. We acknowledge that some proposed subdivisions of CM in the classification system may ultimately be shown to be immaterial and may simply represent differences along a continuum of disease; if so, the schema will have served its purpose in promulgating testable hypothesis and will be revised. However, our proposal outlines the considered opinion of experts that these categories describe distinct and distinguishable clinical phenotypes common among patients with CM. These categories allow for consistent epidemiological and translational studies to be performed. Studies evaluating differences between the proposed groups will aid our understanding of the immunological features underpinning these phenotypes or of the virulence features of the pathogen that allow for its variable degrees of dissemination. These differences will lay the groundwork for future therapeutics and rational-personalized medicine.
